# Supplementary material for: Strong ferromagnetism of g-C3N4 achieved by atomic manipulation
Source: Nat Commun. 2023 Apr 20;14:2278. doi: 10.1038/s41467-023-38012-8 (PMC10119309; doi:10.1038/s41467-023-38012-8)
Supplement: Supplementary file 1 — Supplementary Information [file 41467_2023_38012_MOESM1_ESM.pdf]

Supplementary Information

Strong ferromagnetism of g-C_3N_4 achieved by atomic manipulation

Lina Du[1, #], Bo Gao[1, #], Song Xu[2], Qun Xu[1,2, *]

[1]College of Materials Science and Engineering, Zhengzhou University, Zhengzhou 450052, PR China;

[2]Henan Institute of Advanced Technology, Zhengzhou University, Zhengzhou 450052, PR China.

*Corresponding author. E-mail: qunxu@zzu.edu.cn

[#]L. Du and B. Gao contributed equally to this work.

Table of Contents

Figures of Supplementary Material Characterizations S3

Figures of Supplementary Magnetic Characterizations S10

Figures of Supplementary Computational Investigations S12

Supplementary Tables S14

References S16

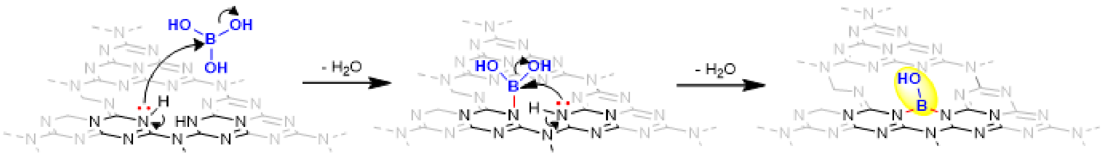

Supplementary Scheme 1. Proposed mechanism of borate decorated 2D g-C_3N_4 nanosheets (B-C_3N_4-X MPa) with in-planar bridging -B(OH)- groups.

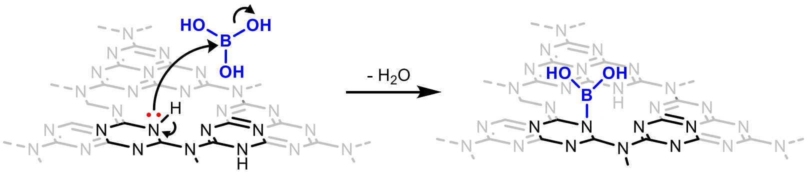

Supplementary Scheme 2. Proposed mechanism of borate decorated 2D g-C_3N_4 nanosheets (B-C_3N_4-X MPa) with terminal -$B(OH)_2$ group.

Figures of Supplementary Material Characterizations

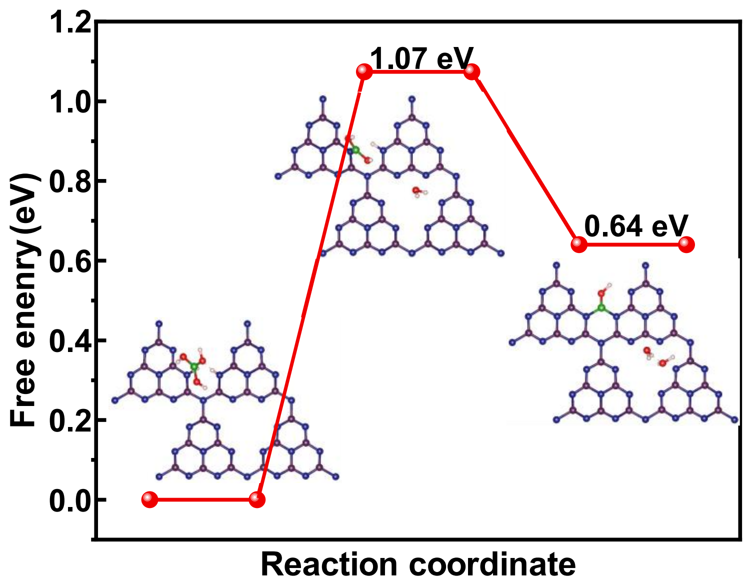

Supplementary Figure 1. The DFT calculated free energy diagram for the formation of -B(OH)n- incorporation.

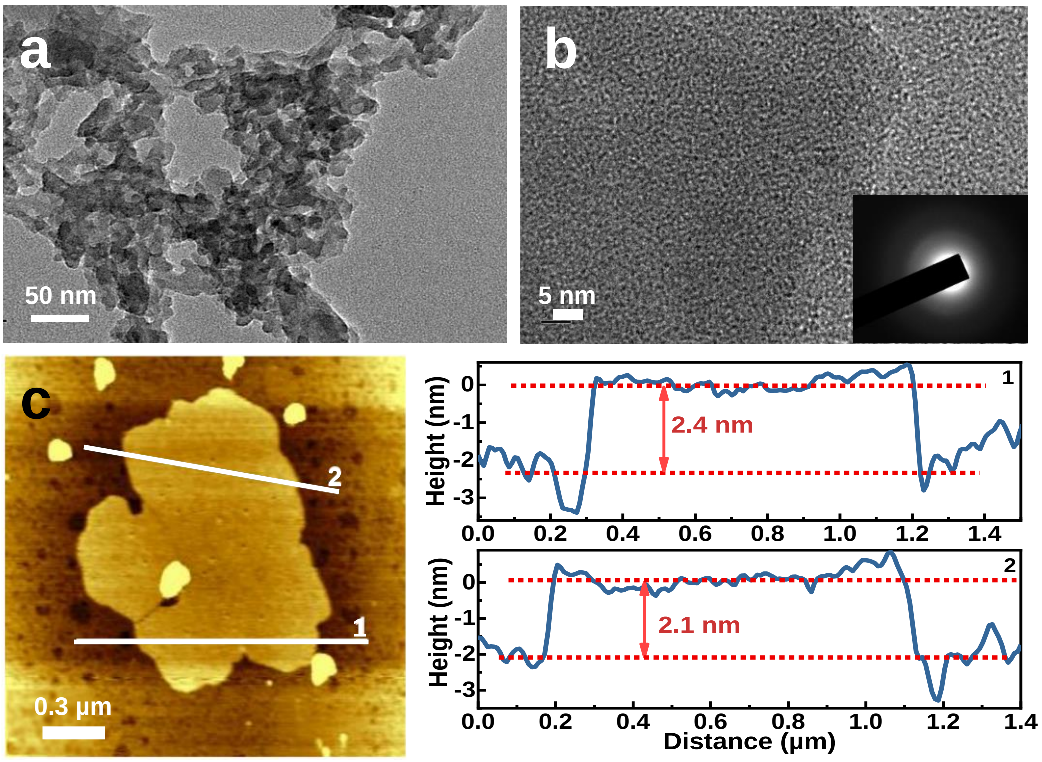

Supplementary Figure 2. (a) TEM and (b) HRTEM images of B-C_3N_4-16 MPa. Inset of (b) is the corresponding SAED pattern. (c) AFM image of B-C_3N_4-16 MPa and the corresponding height image of 1 and 2 lines.

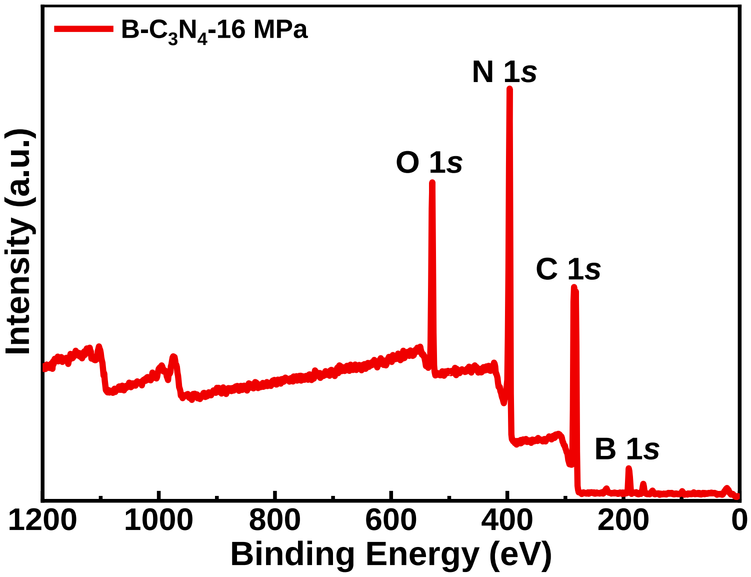

Supplementary Figure 3. XPS survey spectra of B-C_3N_4-16 MPa.

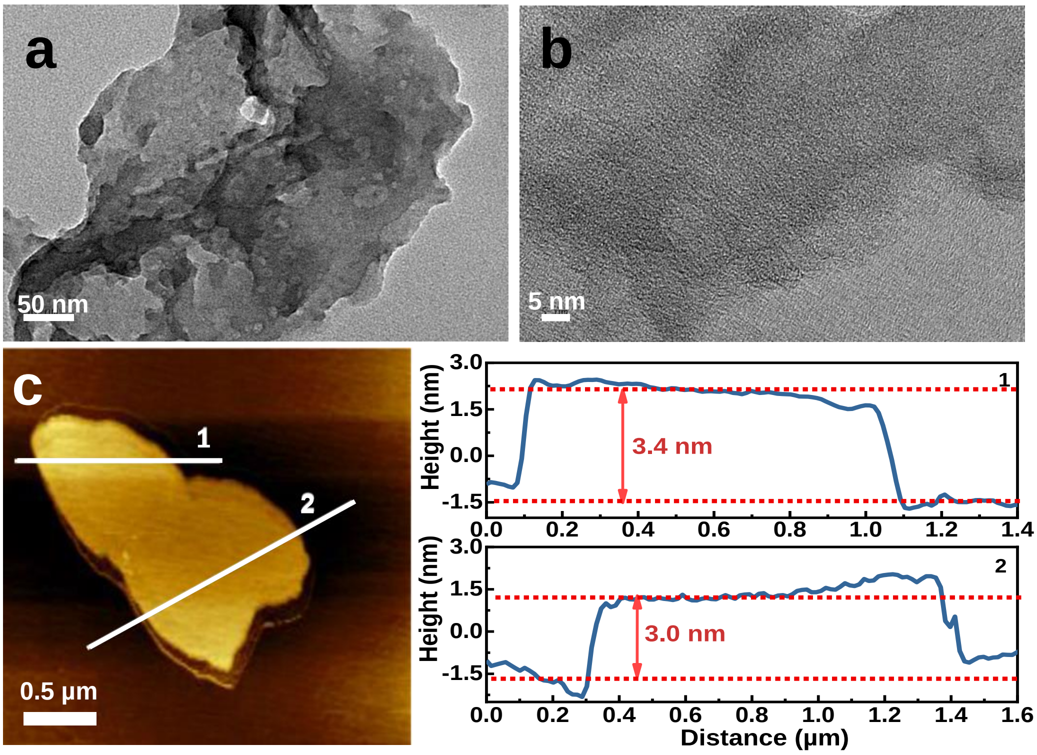

Supplementary Figure 4. (a) TEM and (b) HRTEM images of B-C_3N_4-20 MPa, inset is the corresponding SAED pattern, (c) AFM image of B-C_3N_4-20 MPa and the corresponding height image of 1 and 2 lines.

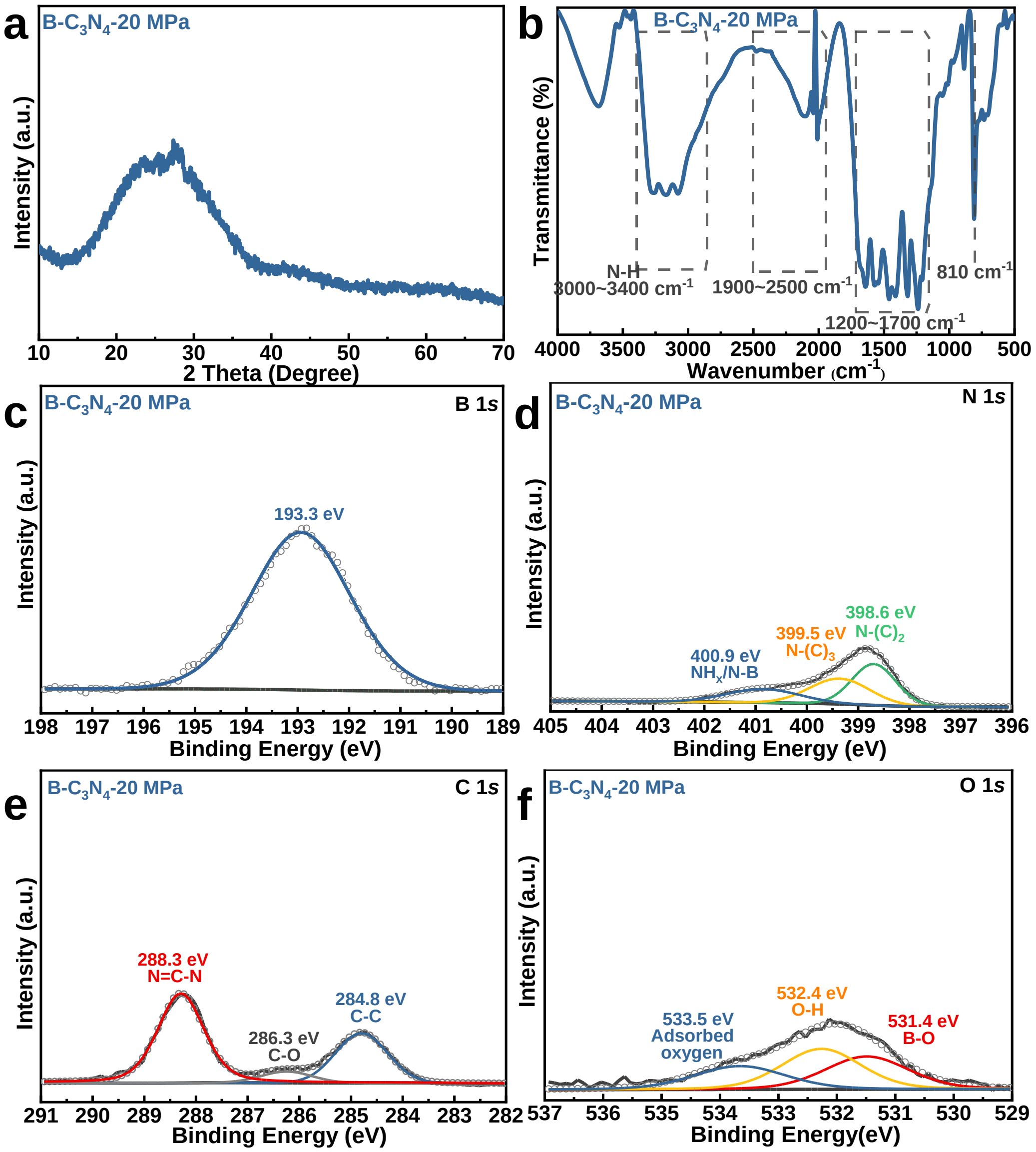

Supplementary Figure 5. (a) XRD pattern of B-C_3N_4-20 MPa. (b) FTIR spectrum of B-C_3N_4-20 MPa;(c) B 1s, (d) N 1s, (e) C 1s and (f) O 1s of B-C_3N_4-20 MPa characterized by XPS.

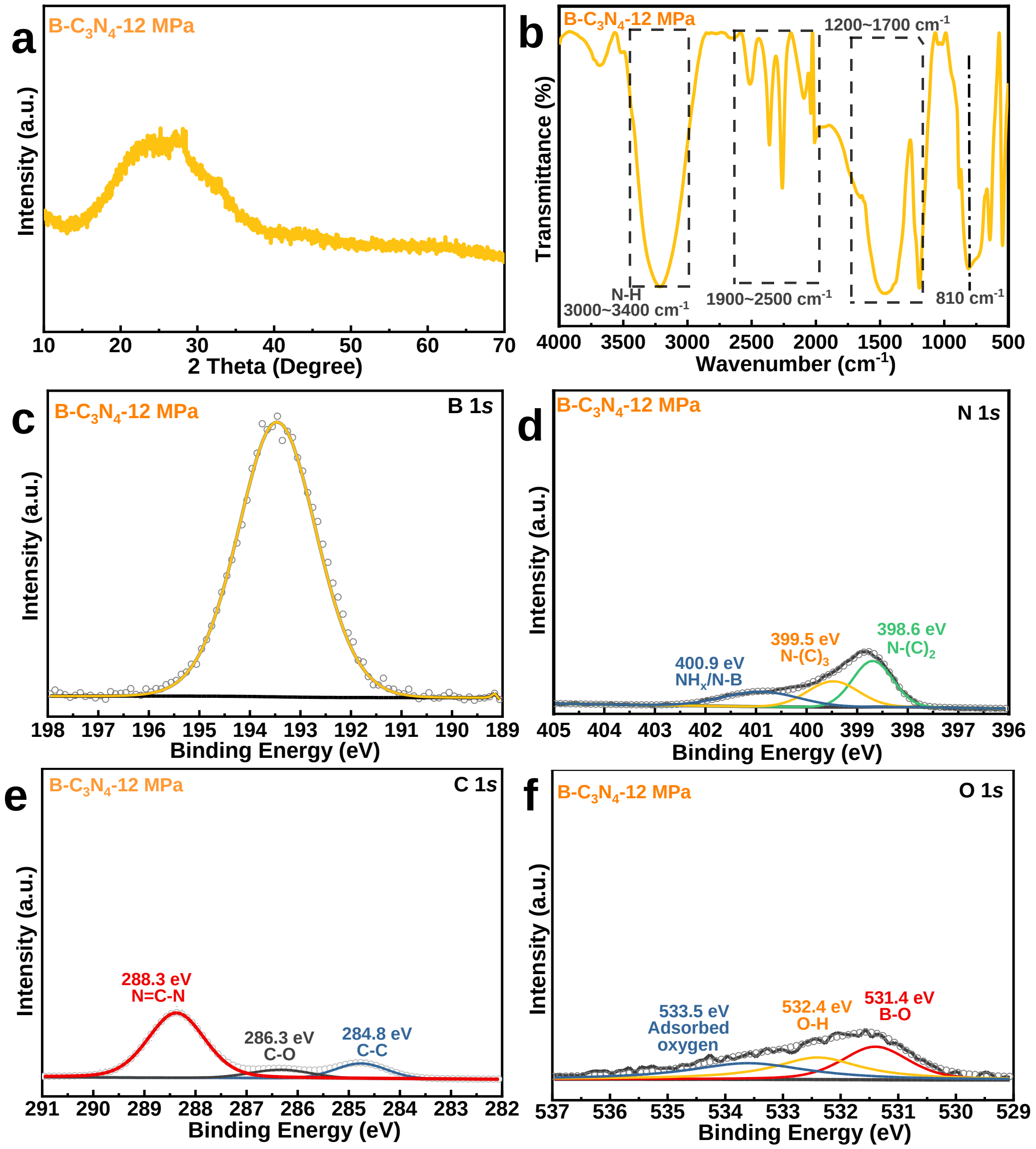

Supplementary Figure 6. (a) XRD pattern of B-C_3N_4-12 MPa. (b) FTIR spectrum of B-C_3N_4-12 MPa;(c) B 1s, (d) N 1s, (e) C 1s and (f) O 1s of B-C_3N_4-12 MPa characterized by XPS.

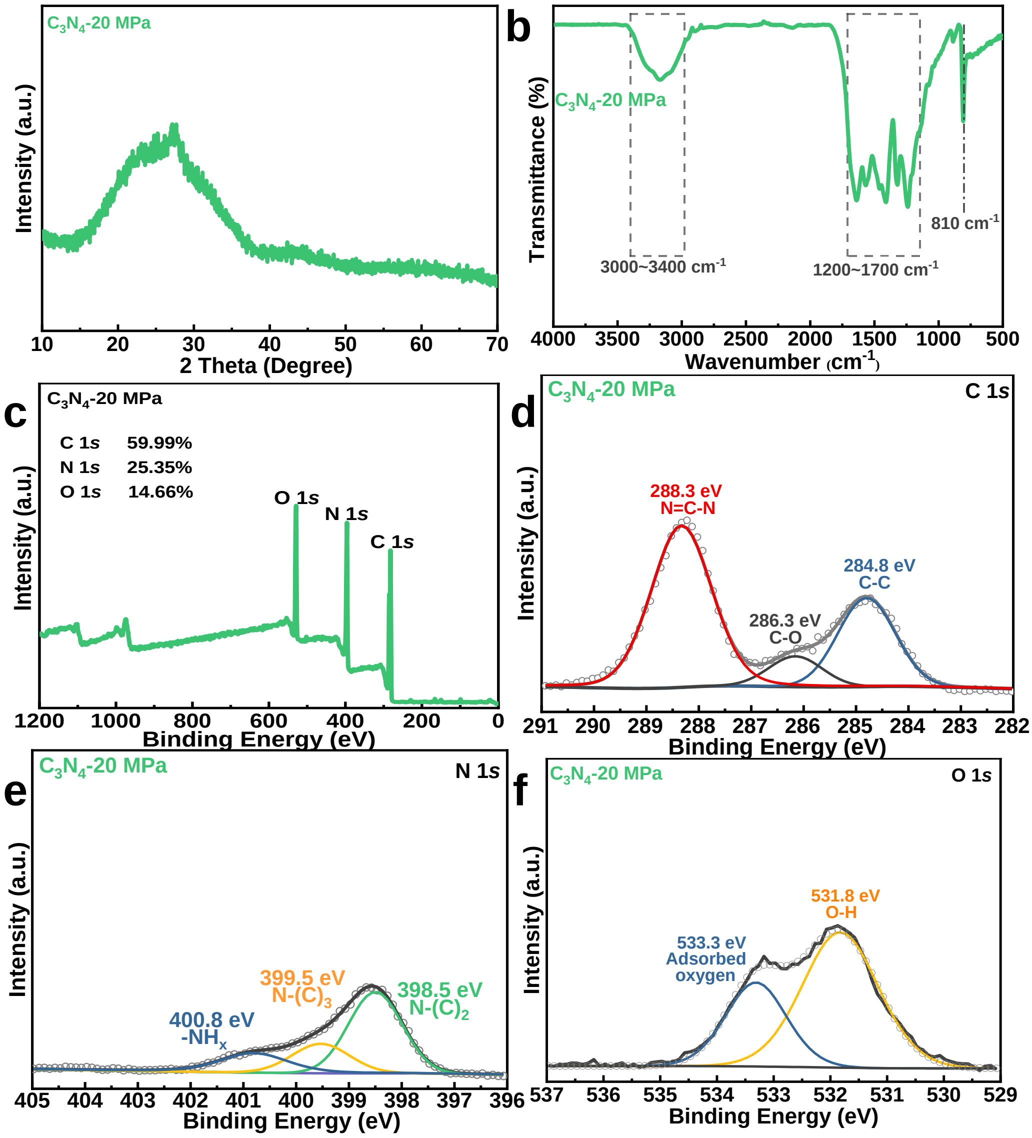

Supplementary Figure 7. (a) XRD pattern of C_3N_4-20 MPa. (b) FTIR spectrum of C_3N_4-20 MPa;(c) XPS survey spectra, (d) C 1s, (e) N 1s and (f) O 1s of C_3N_4-20 MPa characterized by XPS.

Figures of Supplementary Magnetic Characterizations

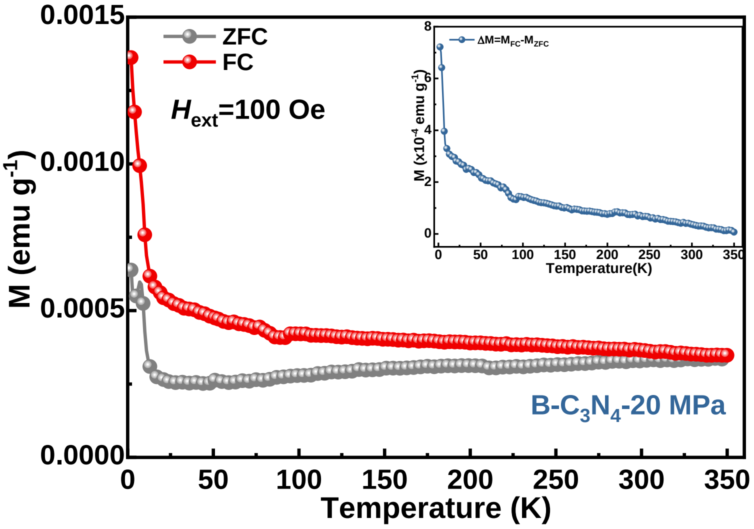

Supplementary Figure 8. FC-ZFC magnetization curve of B-C_3N_4-20 MPa in external magnetic field of 100 Oe. Inset: $\Delta M = M_{FC} - M_{ZFC}$ curve.

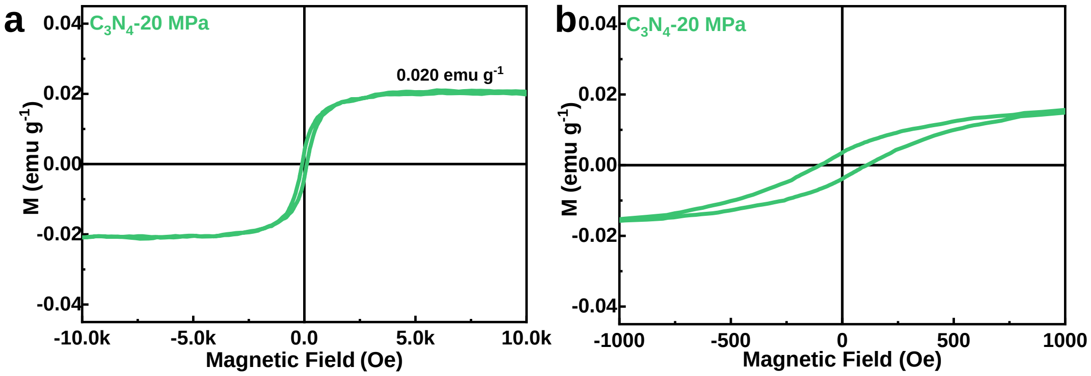

Supplementary Figure 9. (a) M–H curve of C_3N_4-20 MPa at 300 K; (b) The corresponding magnified M-H curves of C_3N_4-20 MPa near H = 0.

Influence of oxygen containing groups on magnetism

Comparing to the -B(OH)- groups, the magnetic contribution from the oxygen containing functional groups introduced to g-C_3N_4 is limited, as suggested by experimental results. Specifically, B-C_3N_4-16 MPa, which contains a lower oxygen but higher boron content, exhibits a significantly stronger *Ms* comparing to B-C_3N_4-20 MPa (0.043 *vs.* 0.026 emu g^{-1}, Table S1). Additionally, the magnetism of B-C_3N_4-20 MPa is significantly stronger to comparing to its analogue without -B(OH)- groups (C_3N_4-20 MPa), where both samples are prepared under identical condition with similar oxygen contents (Figure S7 and S9). Importantly, we propose the -B(OH)- groups boost the magnetism of g-C_3N_4 by establishing long-range magnetic sequence in this work, which is a quite unique mechanism comparing to the magnetism originated from other functional groups including oxygen containing groups.

Figures of Supplementary Computational Investigations

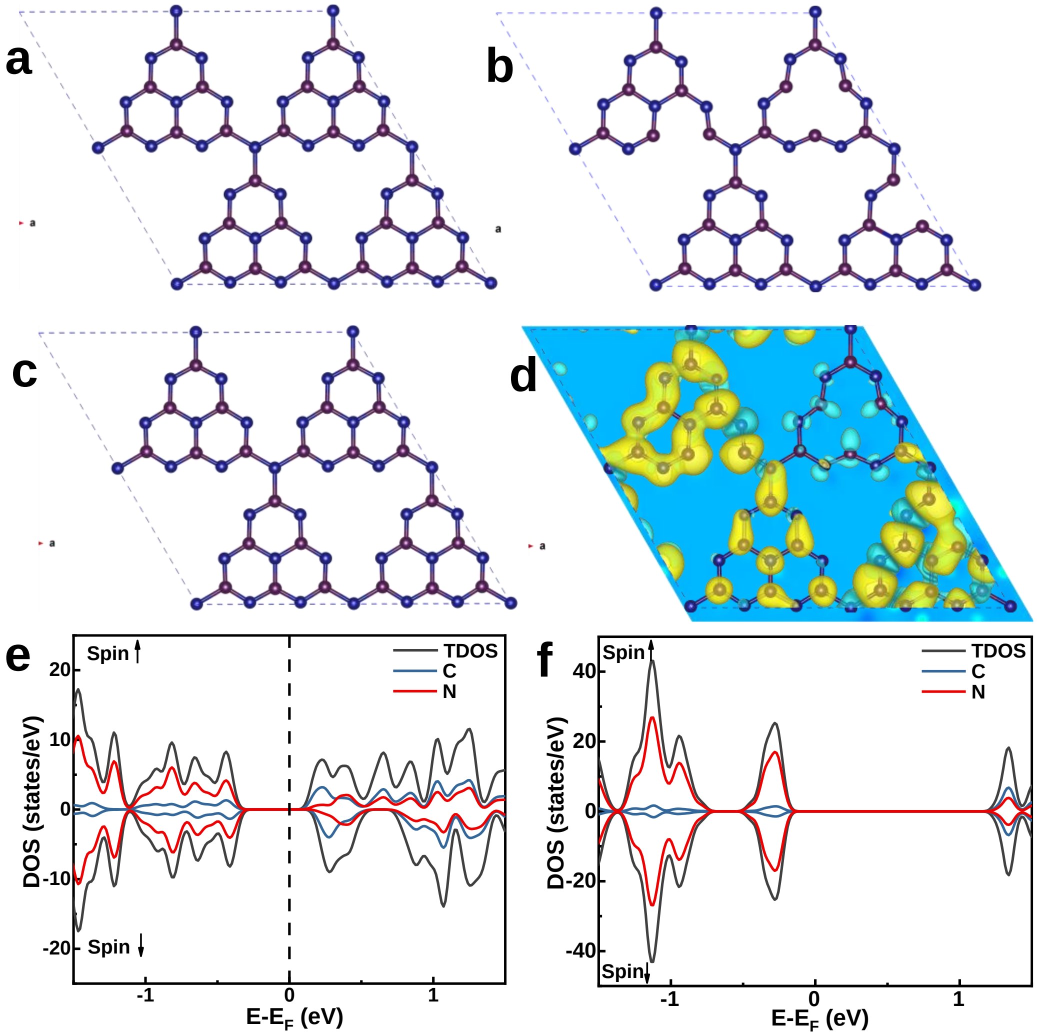

Supplementary Figure 10. **Spin-polarized DFT-calculations for the magnetism of bulk C_3N_4 and g-C_3N_4 with N defects.** The computational structural model of (a) bulk C_3N_4, (b) g-C_3N_4 with N defects. The corresponding top-view of spin charge density of (c) bulk C_3N_4, (d) g-C_3N_4 with N defects, the yellow and blue equipotential profiles represent majority-spin up and minority-spin down, respectively. The corresponding spin resolved total density of states (DOS) and partial density of states (PDOS) for (e) bulk C_3N_4, (f) g-C_3N_4 with N defects.

The effect of N atom loss on the ferromagnetism of g-C_3N_4.

The unit structures of bulk C_3N_4 and g-C_3N_4 (monolayer with N defects) are optimized and the detailed calculations are included in the Figure S10. According to the spin charge density distribution and density of states (DOS), the intact g-C_3N_4 exhibits symmetric total spin-polarized density, indicating no resultant spin polarization and net magnetic moment (Figure S10a, 10c and 10e). In sharp contrast, in the presence of N

defects, the conduction band of g-C_3N_4 is shifted down and the g-C_3N_4 exhibits asymmetric spin-up (majority) and spin-down (minority) occupations near the Fermi energy level (E_F). The charge density distribution is mainly concentrated near the N atoms of the N-C_2 defects, suggesting that the magnetic moment is mainly located on the N atoms with a p-orbital (dumbbell-like) feature (Figure S10b, 10d and 10f). Therefore, it can be concluded that the loss of N atoms in N-C_2 sites are the main ferromagnetic sources.

Supplementary Tables

Supplementary Table 1. Surface compositions of the bulk C_3N_4, B-C_3N_4-12 MPa, B-C_3N_4-16 MPa and B-C_3N_4-20 MPa obtained from the XPS analysis.

	C 1s %	N 1s %	B 1s%	N 1s/C 1s	N-C_2/N-C_3	-NHx%
Bulk C_3N_4	41.39	52.94	-	1.28	2.69	17.16
B-C_3N_4-12 MPa	46.67	42.03	2.53	0.90	1.39	26.27
B-C_3N_4-16 MPa	50.75	35.76	2.84	0.70	1.29	28.30
B-C_3N_4-20 MPa	51.22	31.16	2.27	0.61	1.26	20.26

Supplementary Table 2. Reported RT FM in other carbon materials.

Sample	Magnetic species	M_S (emu g^{-1})	T_C *(K)*	Ref.
Naphthalene–130(N14)	FM	0.022	>300	Carbon **136**, 125-129 (2018) [1]
B-doped g-C_3N_4 nanosheets (B-1.3%)	FM	0.008	>300	Sci. Rep. **6**, 35768 (2016) [2]
Hydroxofluorographene$C_{18}(OH)_{3.4}F_6$	FM	0.0125	>300	ACS Nano **12**, 12847–12859 (2018) [3]
Carbonized polymer dots (CPDs300)	FM	0.021	300	Adv. Sci. 1801192 (2018) [4]
Fe-Graphene quantum dotsolid sheets	FM	0.000349 emu	-	Appl. Surf. Sci. **548**, 149195 (2021) [5]
MoS_2/ graphene heterostructures	FM	0.035	>300	Nano Res. **14**, 4182-4187 (2021) [6]
B-C_3N_4-16 MPa	FM	0.043	550	This work

Supplementary Table 3. The contents of various selected metals in the samples detected by the inductively coupled plasma mass spectrometry (ICP-MS) technique. The unit is ppm, and 'ND' denotes 'Not detected' or the signals are lower than the detected limit (0.01).

Sample	Cr	Mn	Fe	Co	Ni	Cu	Zn
bulk C_3N_4	0.012	ND	0.179	ND	ND	0.055	ND
B-C_3N_4-12 MPa	0.014	ND	0.118	ND	ND	0.214	ND
B-C_3N_4-16 MPa	0.012	ND	0.095	ND	ND	0.550	ND
B-C_3N_4-20 MPa	0.014	ND	0.127	ND	ND	0.654	ND

References

1. Wu, X. L. *et al*. Room temperature ferromagnetism in naphthalene. *Carbon* **136**, 125-129 (2018).
2. Gao, D. Q. Liu, P. T., Si, M. S., Xue, D. S. Atomically thin B doped *g*-C_3N_4 nanosheets: high-temperature ferromagnetism and calculated half-metallicity. *Sci. Report* **6**, 35768 (2016).
3. Jiří, T. *et al*. Zigzag sp^2 carbon chains passing through an sp^3 framework: a driving force toward room-temperature ferromagnetic graphene. *ACS Nano* **12**, 12847-12859 (2018).
4. Lu, S. Y. *et al*. Graphitic nitrogen and high-crystalline triggered strong photoluminescence and room-temperature ferromagnetism in carbonized polymer dots. *Adv. Sci.* **6**, 1801192 (2018).
5. Ganapathi, B. *et al.* Room temperature weakly ferromagnetic energy band opened graphene quantum dot coupled solid sheets – A possible carbon based dilute magnetic semiconductor. *Appl. Surf. Sci.* **548**, 149195 (2021).
6. Cai, L. *et al*. Ultrahigh-temperature ferromagnetism in MoS_2 Moiré superlattice/graphene hybrid heterostructures. *Nano Res.* **14**, 4182-4187 (2021).
