# Supplementary figures and images for: Strong ferromagnetism of g-C3N4 achieved by atomic manipulation
Source: Nat Commun. 2023 Apr 20;14:2278. doi: 10.1038/s41467-023-38012-8 (PMC10119309; doi:10.1038/s41467-023-38012-8)

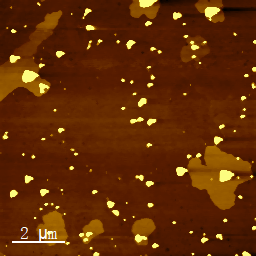

Supplement: Supplementary file 3 — Source Data [file 41467_2023_38012_MOESM3_ESM.zip › NC Source Data/AFM results/B-C3N4-16 MPa.png]
